# A molecular epidemiological investigation of contagious caprine pleuropneumonia in goats and captive Arabian sand gazelle (*Gazella marica*) in Oman

**DOI:** 10.1186/s12917-024-03969-1

**Published:** 2024-04-25

**Authors:** Haytham Ali, Mahmoud El-Neweshy, Julanda Al Mawly, Martin Heller, Michael Weber, Christiane Schnee

**Affiliations:** 1 College of Agricultural and Marine Sciences, Sultan Qaboos University, Muscat, Oman; 2 Department of Pathology, Faculty of Veterinary Medicine, Zagazig University, Zagazig, 44511 Egypt; 3 Department of Pathology, Faculty of Veterinary Medicine, Kafrelsheikh University, Kafrelsheikh, 33516 Egypt; 4 Central Laboratory of Animal Health, Ministry of Agriculture, Fisheries and Water Resources, Muscat, Oman; 5 Institute of Molecular Pathogenesis, Friedrich-Loeffler-Institut, Naumburger Str. 96a, 07743 Jena, Germany

**Keywords:** *Mycoplasma capricolum ssp. capripneumoniae*, Contagious caprine pleuropneumonia, Oman, Whole genome SNP analysis

## Abstract

**Background:**

Contagious caprine pleuropneumonia (CCPP) is a fatal WOAH-listed, respiratory disease in small ruminants with goats as primary hosts that is caused by *Mycoplasma capricolum* subspecies *capripneumoniae* (*Mccp*). Twelve CCPP outbreaks were investigated in 11 goat herds and a herd of captive Arabian sand gazelle (*Gazella marica*) in four Omani governorates by clinical pathological and molecular analysis to compare disease manifestation and *Mccp* genetic profiles in goats and wild ungulates.

**Results:**

The CCPP forms in diseased and necropsied goats varied from peracute (5.8%), acute (79.2%) and chronic (4.5%) while all of the five necropsied gazelles showed the acute form based on the clinical picture, gross and histopathological evaluation. Colonies of *Mccp* were recovered from cultured pleural fluid, but not from lung tissue samples of one gazelle and nine goats and all the isolates were confirmed by *Mccp*-specific real time PCR. Whole genome-single nucleotide polymorphism (SNP) analysis was performed on the ten isolates sequenced in this study and twenty sequences retrieved from the Genbank database. The *Mccp* strains from Oman clustered all in phylogroup A together with strains from East Africa and one strain from Qatar. A low variability of around 125 SNPs was seen in the investigated Omani isolates from both goats and gazelles indicating mutual transmission of the pathogen between wildlife and goats.

**Conclusion:**

Recent outbreaks of CCPP in Northern Oman are caused by *Mccp* strains of the East African Phylogroup A which can infect goats and captive gazelles likewise. Therefore, wild and captive ungulates should be considered as reservoirs and included in CCPP surveillance measures.

**Supplementary Information:**

The online version contains supplementary material available at 10.1186/s12917-024-03969-1.

## Background

Contagious caprine pleuropneumonia (CCPP) is a WOAH-listed, respiratory disease of small ruminants with goats as primary hosts. It is caused by *Mycoplasma capricolum* subspecies *capripneumoniae* (*Mccp*), a member of the *Mycoplasma mycoides* cluster [1]. CCPP causes high morbidity and mortality rates that may reach 100% and 80%, respectively [2, 3]. The disease results in severe economic losses in many countries of Africa, Asia and the Middle East, including Oman [4–7]. The characteristic clinical signs in acute and subacute CCPP are fever, respiratory distress and coughing with postmortem changes confined to the thoracic cavity in form of unilateral severe pleural effusion and fibrinous pleuropneumonia [3, 8].

Although CCPP is considered a goat–specific disease, it has also been clinically described in many wild species either held in captivity or free ranging: Nubian ibex (*Capra ibex nubiana*), wild goat (*Capra aegagrus*), gerenuk (*Litocranius walleri*), *Laristan mouflon* (*Ovis orientalis laristanica*) in Qatar [9], Arabian Oryx (*Oryx leucoryx*) in Saudia Arabia [10], Tibetan antelope (*Pantholops hodgsonii*) in China [11] and Sand gazelle (*Gazella marica*) in UAE [12]. Moreover, *Mccp* has been isolated from sheep in Kenya and Egypt [13, 14].

Mycoplasmal pneumonia was frequently misdiagnosed as CCPP due to the closely related phenotypic and genomic properties of *Mccp* with others pathogenic mycoplasmas in ruminants, such as *M. mycoides* ssp. cap*ri* or *M. ovipneumoniae* [15, 16]. In addition to examination of typical clinical and necropsy signs, isolation and molecular identification of *Mccp* strains are required for confirmation of CCPP. Because *Mccp* is very fastidious and difficult to grow, PCR is the method of choice, and several PCR assays have been proposed for molecular detection [17–20]). Molecular typing tools include multi locus sequence typing (MLST) [21], core genome (cg) MLST [22] and single nucleotide polymorphism (SNP)-based whole genome analysis [23].

The current study aimed to conduct a whole genome SNP analysis on the isolated *Mccp* from several CCPP outbreaks that occurred in goats and captive Sand gazelles in Oman between 2019 and 2020. This should allow the reconstruction of epidemiological relationships between isolates from different locations and from wild and domestic hosts. Diagnosis of CCPP was performed and confirmed through gross and histopathological examination, immunohistochemistry (IHC) antigen localization, bacterial culture, and real time PCR.

## Results

### Case history

A herd of Arabian sand gazelle (*Gazella marica*) in a private farm in Muscat, Oman, suffered from high mortalities of both, adult and young animals in April 2020. The responsible veterinarian reported a shortness of breath in some animals before they were found dead and no other clinical signs were evident. Animals were treated with oral antibiotics and vitamins (Keproceryl®, Afrimash, Holland; Tylosin 20%, MUSCATPHARMA, Oman). Animals did not respond to the treatment and 70 out of 120 died within one week from the onset of clinical signs. Five deceased gazelles were submitted to the Central Veterinary Laboratory (CVL), Ministry of Agriculture, Fisheries and Water Resources, Oman for postmortem examination and laboratory diagnosis. The gazelles had been vaccinated against foot and mouth disease (FMD), *peste des petits* ruminants (PPR), pasteurellosis, theileriosis, enterotoxaemia, and bluetongue disease.

**Fig. 1:**
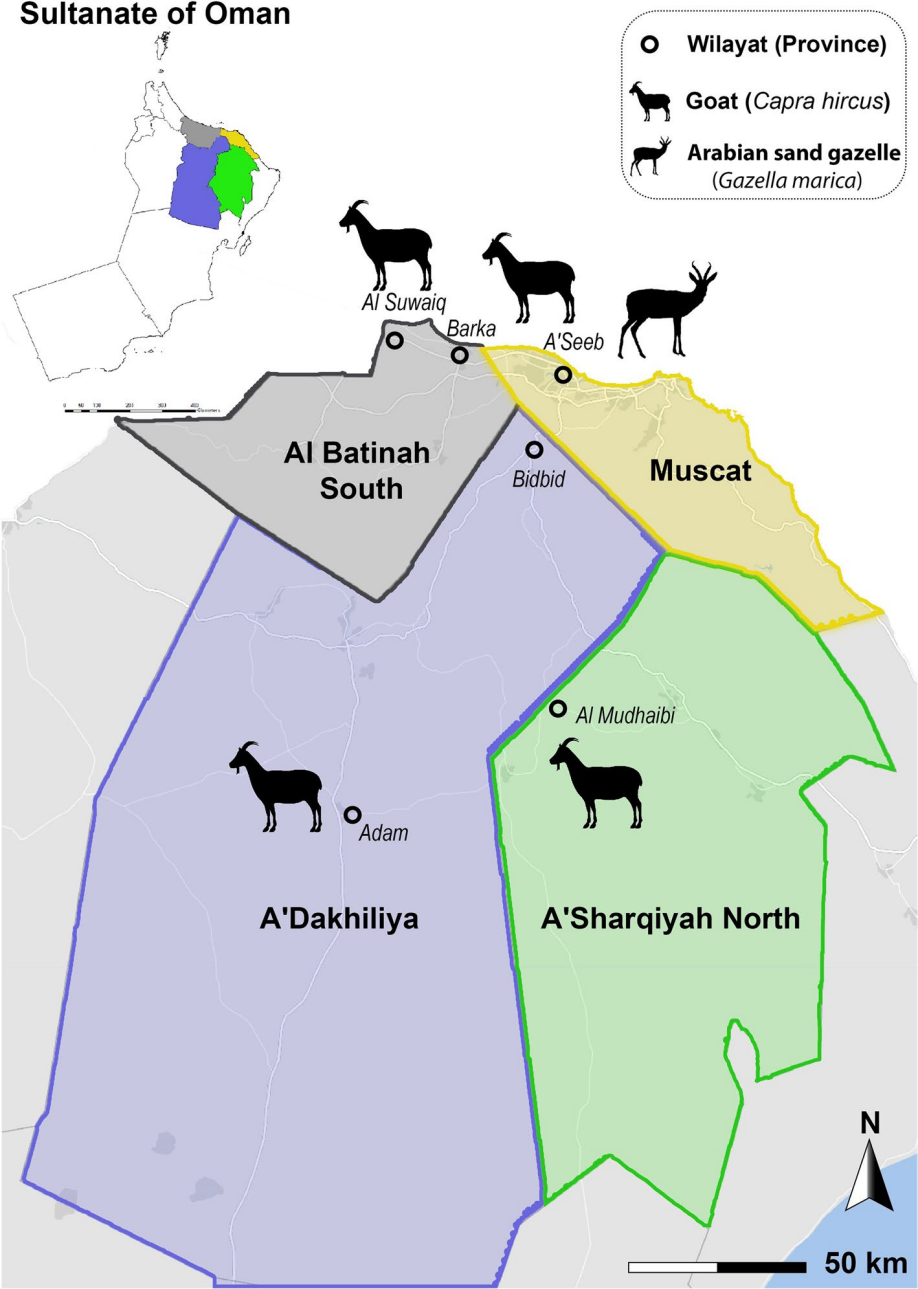
Locations of CCPP outbreaks occurring in 2019/2020 in four Northern governorates of the Sultanate of Oman. (Created by https://www.scribblemaps.com/)

Eleven further CCPP outbreaks were investigated in goat herds from 2019 to 2020 in the four Northern Omani governorates Muscat, A’Sharqiyah, A’Dakhiliyah, and Al-Batinah South (Fig. 1).

The outbreaks appeared in the peracute form where only sudden death without obvious symptoms of few animals was reported and in the acute form where animals showed signs of dyspnea, nasal discharges, followed by either death or recovery. Thirty-four deceased animals were submitted to the Central Veterinary Laboratory, Ministry of Agriculture, Fisheries and Water Resources, Oman for postmortem examination and laboratory diagnosis. No data were available about the vaccination routine for goat farms. All necropsied animals were obtained from private farms and submitted to the CVL by local veterinarians after obtaining oral consent from the owners.

An overview of the farms and cases included in this study is given in Additional Table 1.

### Clinical signs, pathology, histopathology and immunohistochemistry

**Fig. 2:**
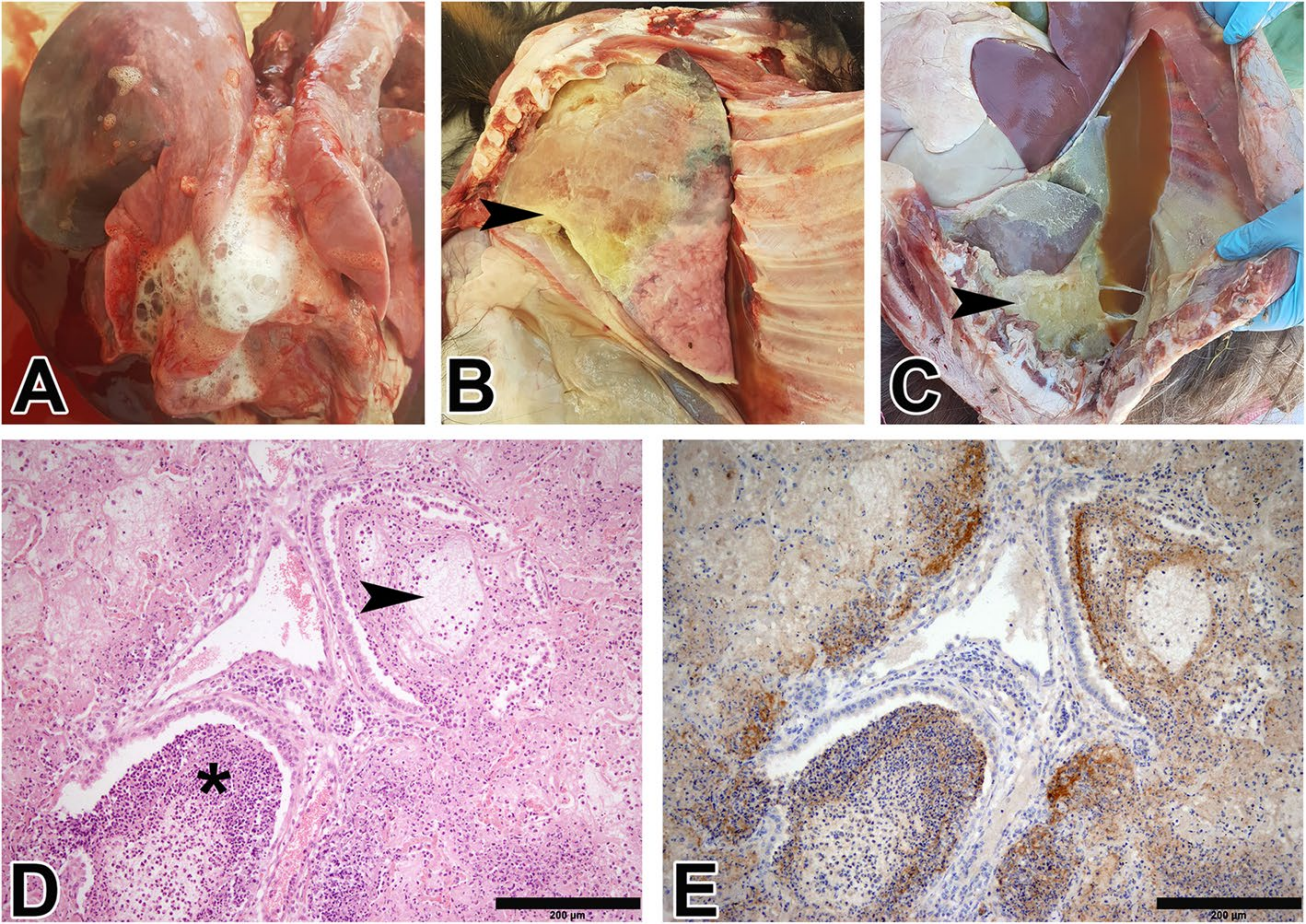
Representative graphs of gross, histopathological and IHC examinations of the necropsied CCPP naturally infected goats; **A** Peracute form represented by lung congestion, severe pulmonary edema and white-colored foamy fluid in the ‎trachea; **B** Acute form showing marbling appearance and hepatization of the lungs, hydrothorax, and fibrinous pleuritis **C** Chronic form characterized by unilateral pleural adhesion (arrowhead), lung hepatization and severe hydrothorax; **D** Photomicrograph of pulmonary tissue showing fibrin threads deposition in a bronchiole (arrowhead) and surrounding alveoli besides obliteration of another bronchiole with inflammatory cells (asterisk) (H&E, bar=200µm); **E** Consecutive section of (**D**) stained with anti-*Mccp* antibody showing intense brown positive staining in the fibrin deposits, inflammatory cells obliterating the bronchioles and alveoli, and the peribronchial lymphoid aggregates (DAB staining, Hematoxylin counterstaining, bar=200µm)

The recorded clinical signs, post-mortem and histopathological lesions of different forms of CCPP in goats and Arabian sand gazelles are summarized in Additional Table 2. In goats (Fig. 2A-D), pulmonary edema were the only recorded lesions in the peracute form of CCPP evidenced by the frothy fluid in the trachea and the lungs cut sections (Fig. 2A). Microscopically, the pulmonary alveoli were filled with homogenous eosinophilic substance admixed with polymorphonuclear cells. In the acute form, post-mortem examination of the lungs showed unilateral fibrinous pleuropneumonia, marbling appearance and hepatization of the lungs, hydrothorax, and fibrinous pleuritis (Fig. 2B). Histopathology revealed marked proteinaceous fibrin material deposition in the pulmonary alveoli, alveolar hemorrhage and edema, interstitial edema, and pulmonary capillary congestion. The majority of the bronchi and bronchioles were filled with fibrin deposits and acute inflammatory cells mainly neutrophils (Fig. 2D). In chronic form, unilateral pleural adhesion, lung hepatization and severe hydrothorax were observed upon necropsy (Fig. 2C). Histologically, multifocal necrotic areas in the lungs and fibrin deposits admixed with aggregations of macrophages in the alveolar spaces and bronchi were observed.

**Fig. 3:**
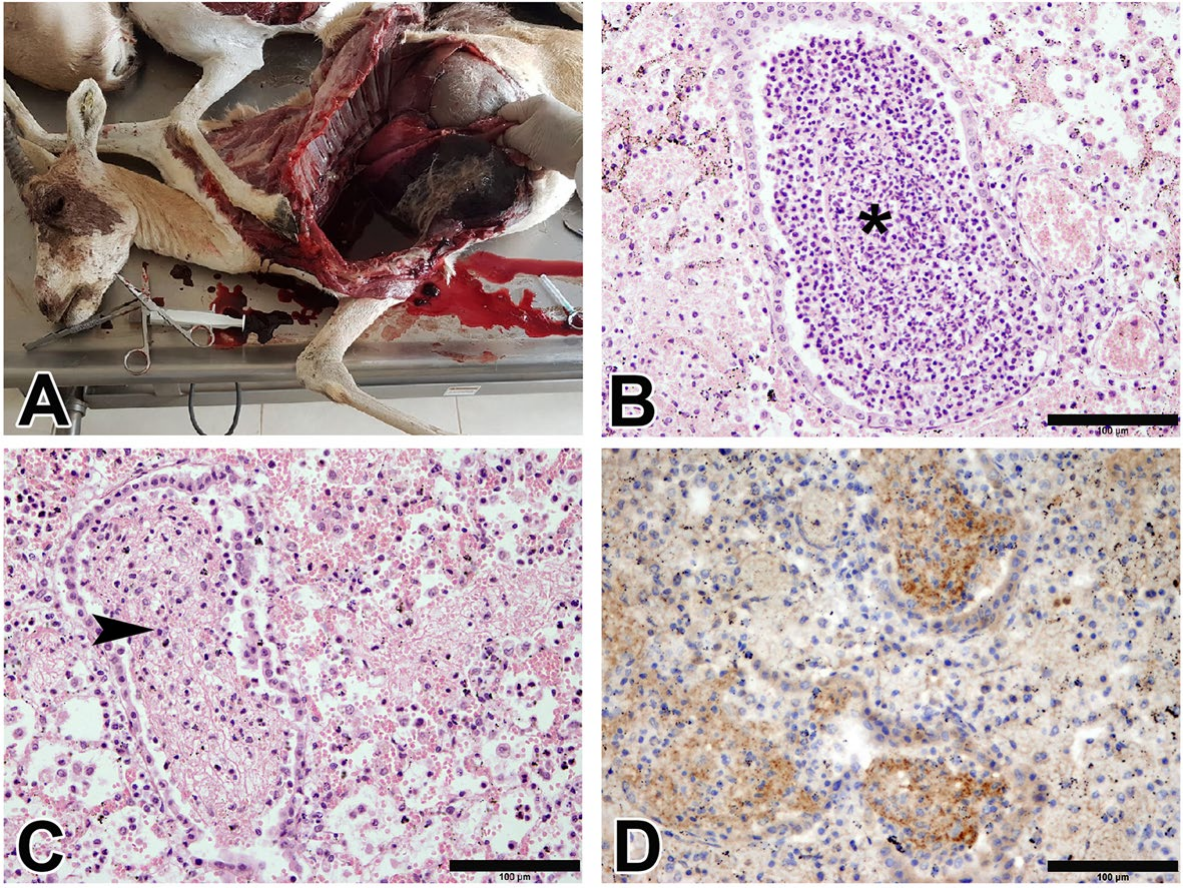
Representative graphs of gross, histopathological and IHC examinations of the necropsied CCPP naturally infected Arabian sand gazelle. **A** Acute form characterized by unilateral pleural adhesion (arrowhead), lung hepatization, and severe hydrothorax; **B** Lung showing a bronchiole completely obliterated with macrophages and few neutrophils (asterisk) (H&E, bar = 100µm); **C** fibrinous deposition and inflammatory cells obstructing a bronchiole (arrowhead) (H&E, bar = 100µm); **D** Immunostaining of lung tissue section with anti-*Mccp* antibody showing intense brown positive staining in the fibrin deposits, and inflammatory cells obliterating the bronchioles and alveoli (DAB staining, Hematoxylin counterstaining, bar = 100µm)

Necropsied gazelles (Fig. 3A-C) exhibited an acute form of the disease evidenced by unilateral pleural adhesion, lung hepatization, and severe hydrothorax. Microscopically, the pulmonary tissues showed completely obliterated bronchioles neutrophils, macrophages and fibrinous deposition.

Detection of *Mccp* using immunohistochemistry in goats and gazelles revealed a diffuse positive reaction in the fibrin deposits and inflammatory cells that were obliterating the bronchioles and alveoli. Intense positive staining was detected in the peribronchial lymphoid aggregates as well (Figs. 2E, 3D).

### Detection of *Mccp* DNA in clinical samples

In total, 29 animals (one gazelle and 28 goats) from eleven different herds, all with acute symptoms of CCPP were examined by means of real time PCR detection of *Mccp* with a predetermined cut off for positivity at a Cq value of 38 in 24 lung, 13 pleural fluid and 5 nasal swab samples (see Additional Table 3). All lung and pleural fluid samples were shown to be *Mccp*-positive with high bacterial loads represented by low Cq values with a mean of 18.9 and 19.8, respectively. On the other hand, in nasal swabs from symptomatic animals, high Cq values (33–45) were detected and only 3 of 5 samples were assigned *Mccp*-positive.

### Isolation of *Mccp*

**Fig. 4:**
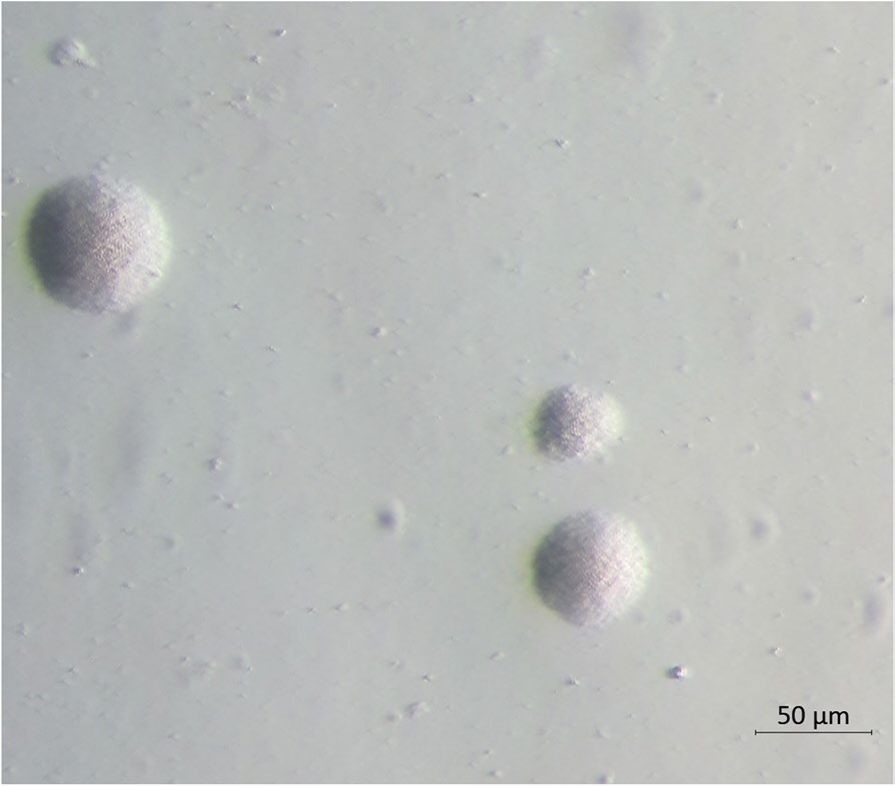
Colonies of *Mycoplasma capricolum* ssp. *capripneumoniae* isolated from the pleural fluid of an Arabian sand gazelle appear on agar plates with MS medium (Mycoplasma Experience) in an unusual centerless form (bar = 50µm)

Isolation of *Mccp* was successful only from pleural fluid, but not from lung tissue of the investigated gazelle carcass although bacterial load of the two sample types was similar as determined by quantitative real time PCR. The obtained isolate 20DL0191 presented with typical *Mycoplasma* colonies on agar plates, with a diameter of 10–50 µm, but without the fried-egg morphology often observed in *Mccp* isolates. (Fig. 4).

Further nine *Mccp* strains were isolated from goats originating from four farms in A’Seeb Muscat governorate (20DL0058, 20DL0072, 20DL190 and 20DL192), the area of the gazelle farm, and from two farms in two other governorates (20DL0060, 20DL0066, 20DL0070, 20DL0194 and 20DL0195). These isolates were also recovered exclusively from pleural fluid, but not from lung tissue.

### Species verification of isolates

The identity of Mycoplasma isolates from the gazelle and from goats was confirmed by *Mccp*-specific real time PCR. All DNA extracts from broth culture tested *Mccp*-positive with Cq values in the range of 18–20.

### Molecular genotyping of Mccp isolates

**Fig. 5:**
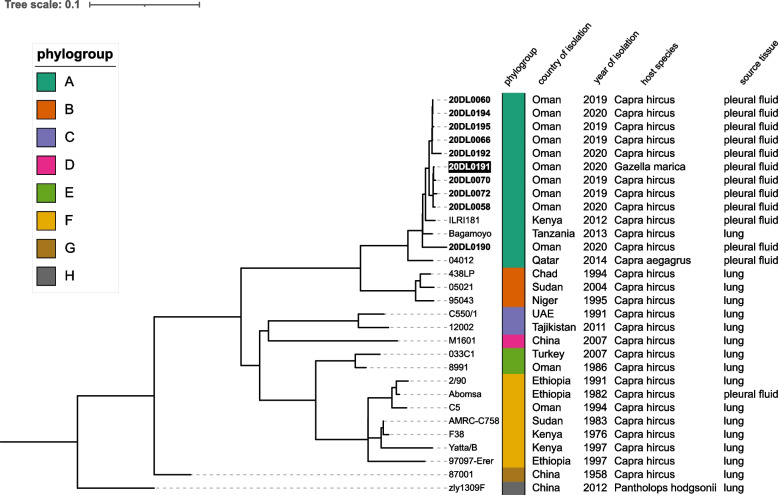
Whole genome SNP-based phylogenetic tree. The tree was constructed by analyzing Illumina reads from one gazelle (highlighted white on black background) and nine goat (in bold) *Mccp* strains from Oman as well as twenty international *Mccp* genomes currently available at Genbank. The maximum parsimony tree is based on 2991 core SNPs and was estimated by using FastTree. Tree visualization was done using iTOL. Phylogroups A-H were inferred from Dupuy et al. and Loire et al. [22, 23]

**Fig. 6:**
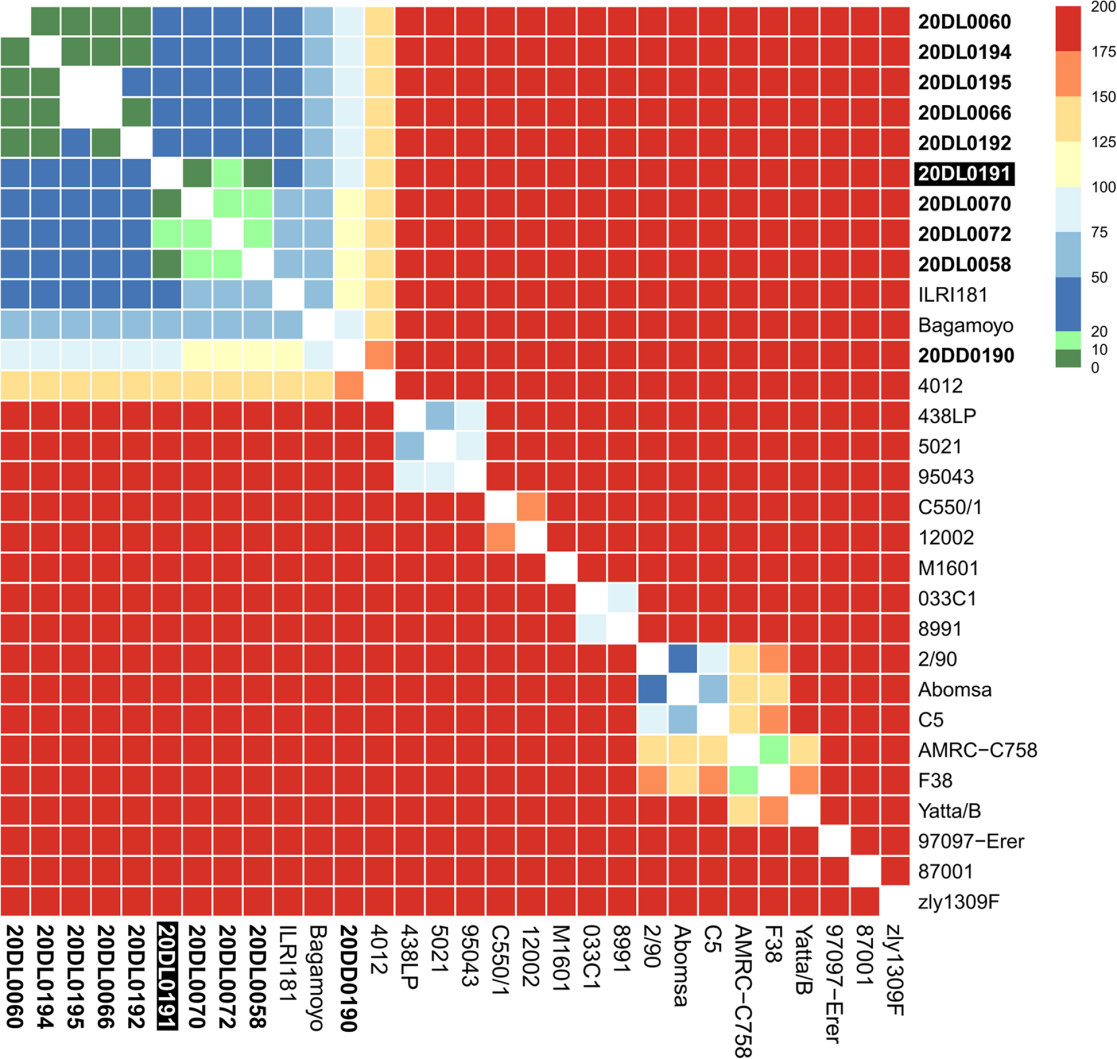
Heatmap of pairwise SNP distances of ten *Mccp* isolates (in bold) recently isolated from a gazelle and goats in Oman and twenty historical isolates of worldwide origin

To elucidate the phylogenetic relationship among the isolate from a devastating CCPP outbreak in Arabian sand gazelles and isolates from local outbreaks in nearby goat flocks as well as to compare these Omani *Mccp* isolates with recent and historic strains world-wide, whole genome-SNP analysis was performed on ten isolates sequenced in this study and twenty sequences retrieved from the Genbank database (see Additional Table 4). In total, 3399 SNPs were identified, while 2991 were assigned core SNPs that occur in all strains. The inferred tree distributes sequences into eight clades showing some correlation with geographic origin. The gazelle isolate 20DL0191 clusters together with all nine recent goat isolates from Oman in phylogroup A. The closest relatives are isolates 20DL0058, 20DL0070 and 20DL0072 with less than 20 SNPs distance (Figs. 5 and 6). They originate from two goat farms in the same region (A’Seeb Muscat) and from one farm in the neighbouring Bidbid—A’Dakhiliyah governorate. Goat isolates 20DL060, 20DL066, 20DL0194 and 20DL0195 were obtained from animals on one farm in Al-Batinah South governorate and seem to be clonal with less than 10 SNPs. They group in another sub-clade with isolate 20DL0192 from a farm in A’Seeb, Muscat. Isolate 20DL0190 from another goat farm in A’Seeb, Muscat is most distantly related to all recent isolates from Oman and forms its own sub-clade with 93–106 SNPs with the other recent isolates from Oman. The closest so far known relatives to all isolates from this study are two goat strains from East Africa, ILRI181 and Bagamoyo, whereas another strain from wildlife on the Arabic peninsula (Qatar) also clusters in phylogroup A. Interestlingly, two historic goat isolates from Oman (8991, 1986 and C5, 1994) belong to different phylogroups (E and F, respectively) and show 769–807 and 867–904 SNPs with the recent isolates.

## Discussion

With an estimated population of 2.1 million, goats are the most abundant livestock and represent an integral part of the farming system in Oman [24]. Contagious caprine pleuropneumonia constitutes a major threat to caprine livestock and thus to the agricultural economy of the country. Recently, an extensive seroepidemiological investigation with risk factor analysis was conducted in 510 small ruminant flocks in Oman revealing seroprevalences of 28.0% in goat flocks and 13.1% in sheep flocks [5]. The current study was initiated following a devastating outbreak of CCPP in a herd of captive Sand gazelles in Muscat governorate. It was designed to compare the infection in local wildlife and goats in terms of disease manifestation, pathology and *Mccp* genotypes in order to clarify a suspected link between the cases.

The rates of morbidity and mortality in the investigated goat herds varied between 2 and 100% and 1 and 50%, respectively, whereas these rates were 100% and 58% in the sand gazelle herd. The relatively higher impact of CCPP on herd health and survival of animals in the wildlife farm could be attributed to different levels of past exposure to *Mccp*. It is believed that the mortality progressively decreases in endemic herds because CCPP develops usually only in naive animals [25]. The gazelles on Farm A might have been exposed for the first time showing higher mortality. Also Lignereux et al. noted a high mortality of about 70% in an affected Sand gazelle herd in UAE [12].

In this study, out of the 34 necropsied goats, the CCPP forms varied from peracute (5.8%), acute (79.2%) and chronic (4.5%) based on the clinical picture, gross and histopathological evaluation. However, all necropsied gazaelles, which were collected from the same outbreak, showed a picture of the acute CCPP form. The predominant acute form in all necropsied goats and gazelles was characterized by the pathognomonic unilateral fibrinous pleuropneumonia with pleural effusion in agreement with the previously described lesions in literature [12, 25]. An intense immunostaining of Mccp was observed in the fibrin deposits and inflammatory cells obliterating both the bronchioles and alveoli in addition to the peribronchial lymphoid aggregates in caprine tissues. In comparison, gazalle lung tissues exhibited a less intense immunostaining of the *Mccp* antigen in the aggregates of neutrophlis and macrophages in agreement with [9] who reported similar findings in infected wild ungulates. This could be attributed to the rapid onset of the disease in gazelles compared to goats probably due to a comporomised immue response in the former.

In many countries, the presence of CCPP was suspected for a long time, but confirmation has been difficult due to the fastidious nature of the pathogen impairing its isolation in pure culture [25]. With the advent of molecular detection by PCR, the diagnostic situation has improved. Using a Taqman-PCR protocol [20], we could detect high *Mccp* loads in all examined lung and pleural fluid samples from deceased animals. The confirmation of the disease from nasal swabs of clinically affected animals was much less reliable with high Cq values or negative results in the PCR. Despite the high DNA load in lung and pleural fluid samples, isolation was successful in only 10 of 37 samples. The exclusive isolation of the agent from pleural fluid, but not from lung tissue was probably due to a stronger contaminating microflora in lung samples which interfered with mycoplasma cultivation. The long transport of the samples from Oman to Germany with an interrupted cold chain may also have had a negative effect on the isolation frequency.

Our straightforward whole genome-based genotyping approach involved SNP calling as suggested by Loire et al. [23], but without the use of reference genomes. We applied it to the ten *Mccp* isolates from this study together with a set of strains representing the global distribution of CCPP whose sequences were available at Genbank (in March 2022). The high number of discovered core SNPs (2991) enabled a high-resolution typing and robust reconstruction of phylogenetic relationships between closely related strains with a local and/or temporal context, but also between isolates from different countries and continents. The inferred tree with phylogroups A to H reflected the overall topology known from classical MLST-based [21], cgMLST-based [22] or core genome-based phylograms [23]. The recent *Mccp* strains from Oman all cluster in phylogroup A together with strains from East Africa and one strain from Qatar. However, considering historic isolates from the region, the Arabian Peninsula exhibits a mosaic of *Mccp* genotypes from phylogroups A, C, E and F. This is not surprising, since Oman, Saudi Arabia and UAE regularly import live goats and sheep from other CCPP endemic regions of Southern Asia, Africa and Turkey [5]. It would be interesting to see if this historic diversity of genotypes is still present or whether individual, particularly successful genotypes have prevailed, as has been observed, for example, with *Mccp* strains from phylogroup A in Tanzania [23].

The variability among the investigated Omani isolates is low with no more than 125 SNPs. Four isolates from Farm 1 were clonal with less than 10 SNPS, whereas isolates from different farms showed slightly higher variability with 10 to 100 SNPs. The impact of spatial distribution within Oman on the genetic variability could not be analyzed in more detail because all isolates came from a limited area in the north of the country.

The observation of Loire et al. [23], that four *Mccp* genome sequences obtained from wildlife on the Arabian Peninsula clustered together in a subclade of phylogroup A, led them to suspect some sort of adaptation to the wild host. Considering the results of our study, this hypothesis is not tenable, because we could prove the close phylogenetic relatedness between the gazelle strain 20DL0191 and strains isolated from goats in neighbouring flocks (e.g. strains 20DL070 and 20DL058) indicating mutual transmission of the pathogen between wildlife and goats. Lignereux et al. [12] also considered the long-distance transmission of infectious droplets from an external goat farm, without direct animal contact the most plausible explanation for the contamination of a gazelle flock in UAE. This implicates that wild ungulates or animals kept in zoos or wildlife parks could develop into reservoirs and represent a risk of CCPP (re-)introduction into goat flocks.

## Conclusion

This study demonstrates that whole genome-based SNP analysis with its high resolution is a valuable tool to dissect the dynamics of local *Mccp* transmission as well as to trace its global epidemiology. Recent outbreaks of CCPP in Northern Oman are caused by *Mccp* strains of the East African Phylogroup A which can infect goats and captive gazelles likewise. Therefore, wild and captive ungulates should be considered as reservoirs and included in *Mccp* surveillance measures.

## Methods

### Pathology

Thirty-four goats and five adult Arabian sand gazelles (2 males and 3 females) were necropsied and samples from the lungs, pleura, trachea and pleural fluid were collected. Lung samples and pleural fluid were kept at – 80 °C and shipped on dry ice to Friedrich-Loeffler-Institut (FLI), Germany for microbial culture and molecular investigations. The other tissue parts were fixed in 10% neutral buffered formalin for 24 h and the specimens were routinely processed for hematoxylin and eosin (H&E) staining [26]. Consecutive sections were immunohistochemically stained using indirect immunoperoxidase staining using ImmPRESS Reagent kit, MP-7800 (Vector Laboratories, Ltd.). Tissue sections were deparaffinized in Shandon Xylene Substitute (Thermo Scientific™), dehydrated in 100% ethanol, and slides were incubated in 10 mM Tris buffer (pH 9.0) (H-3301, Vector Laboratories, Ltd.) at 95°C for 30 min for antigen retrieval. Endogenous peroxide activity was eliminated by treatment with Peroxide Blocking Reagent (Biolegend®). After blocking of non-specific immunoreactives using the ImmPRESS reagent with 2.5% normal horse serum (Vector Laboratories, Ltd.), slides were incubated with polyclonal anti-*M. capricolum* subsp. *capripneumoniae* antibodies from rabbit (BioGenes GmbH, Berlin, Germany) at 1:3000 dilution for 2 h at room temperature in a dark chamber. Sections were washed with buffer and then incubated with ImmPRESS polymer anti-rabbit IgG reagent for 30 min at room temperature. Immunoreactivity was visualized using 3,3′-diaminobenzidine for 5 min and sections were counterstained with hematoxylin for 5 min at room temperature. The slides were examined under the microscope (Olympus B51X microscope; Olympus DP70 camera, Olympus Corporation, Japan) and positive signals appeared as brown color. In negative control slides, the primary antibody was omitted, and slides from a *Mccp* PCR positive goat was included as a positive control.

### Bacterial culture

Tissue samples from pleuro-pneumonic lung lesions (25 mg) and pleural fluids (0,1 mL) were cultured in mycoplasma specific liquid medium containing a phenol-red pH indicator (Mycoplasma Experience Ltd, UK) at 37 °C and 8% CO_2_ under static conditions for 4 to 7 days (until color change of the liquid broth to orange or yellow was observed). Penicillin G (WDT, Garbsen, Germany) was added (1000 IU/ml) to suppress other bacteria. In addition, agar plates with MS Solid Media, (Mycoplasma Experience Ldt, UK), were seeded. From these plates pieces with colonies were transferred to liquid medium when direct cultivation in liquid medium was not successful.

### Nucleic acid extraction

DNA extraction from broth culture, lung tissue, pleural fluid and swab samples for subsequent PCR testing was done using High Pure PCR Template Preparation-Kit (Roche Deutschland Holding GmbH, Mannheim, Germany). To this end, two to four mL of broth culture were centrifuged at 13.000 × g at 6°C for 30 min; the pellet of bacteria was washed once with PBS and then incubated with 200 µL PBS and proteinase K. 50 mg lung tissue or swabs were incubated with lysis buffer and proteinase K provided in the kit and 200 µL pleural fluid was incubated with binding buffer and proteinase K. All subsequent steps were identical for the four different matrices and conducted according to the instruction manual of the DNA preparation kit.

Total DNA extraction for whole genome sequencing was done using Qiagen Genomic DNA Preparation Kit (Qiagen, Hilden, Germany). 100 mL broth culture with approx. 10^9^ CFU in total were centrifuged at 16.000 × g at 6 °C for 30 min. The pellet of bacteria was washed with PBS and resuspended in 1 mL of the recommended buffer of the preparation kit. RNAse (Qiagen, Hilden, Germany) was added to a final concentration of 200 µg/mL and Proteinase K (Qiagen, Hilden, Germany) with a total amount of 900 µg followed by an incubation step for 30 min at 35°C with shaking. Next steps were conducted according to the instruction manual. The final DNA extracts were recovered in 50 µL buffer and checked qualitatively and quantitatively by means of Nanodrop spectrophotometer (Thermo Fisher Scientific, Medison, USA) and QUBIT 2.0 fluorometer (Life Technologies Holdings PTE Ltd, Singapore) as well as agarose gel electrophoresis.

### *Mccp* TaqMan PCR

The real time PCR for the specific detection of *Mccp* was adapted from Settipally et al. [20] and validated in the FLI Mycoplasma Laboratory, Germany. QuantiTect Multiplex PCR Master Mix (Qiagen) was used in a total volume of 15 µL with 800 nM of each primer (*Mccp*-fwd: TTTTTCAAGTGCAAACGACTATG, *Mccp*-rev: TGACTTGGGTGTTAGGACCA), 400 nM probe with LNA ( +) (*Mccp*-pr: FAM-CGGATAG + AACAATA + GCTTTTACAGA-BHQ1) and 2 µL template DNA. To test for PCR inhibition in DNA preparations, an internal amplification control was integrated in duplex PCR runs: 400 nM of each primer (EGFP-1F: GACCACTACCAGCAGAACAC, EGFP-10R: CTTGTACAGCTCGTCCATGC) and 200 nM of probe (EGFP-HEX: HEX-AGCACCCAGTCCGCCCTGAGCA-BHQ1) were used together with 500 copies of a plasmid template per reaction (Intype IC-DNA, Indical Bioscience, Leipzig, Germany) to generate and detect a 177 bp amplicon [27]. The samples were tested in duplicate on a Bio-Rad CFX96 instrument (Bio-Rad, Feldkirchen, Germany) with the following cycling conditions: 95 °C for 10 min and 45 cycles with 95 °C for 30 s and 60 °C for 1 min. A mean quantification cycle (Cq) value of < 38 was considered positive. A 100 GE/µl equivalent of *Mccp* type strain F38 DNA was used as positive control and water instead of template DNA as negative control.

### Whole genome sequencing and SNP analysis

2–10 µg of genomic DNA of each isolate was sent to GATC/Eurofins Genomics (Konstanz, Germany) for genomic library preparation and Illumina MiSeq 2 × 150-bp paired-end sequencing with 5 M read pairs resulting in an average coverage of around 750x. Raw sequencing data were quality-controlled using FASTQC (v0.11.9) and then de novo assembled by applying the Shovill pipeline (v1.0.4) using the SPAdes assembler (v3.15.2). Resulting contig fasta files were annotated using Prokka (v1.14.6). All generated sequencing data have been deposited in the National Center for Biotechnology Information (NCBI) under the accession number PRJNA939501. Additionally, genome assembly data from all available *Mccp* strains on the NCBI GenBank database (21) were downloaded in fasta format. Phylogenetic relationship of the 30 strains was reconstructed based on SNP calling using the software kSNP v3.0 [28], and a maximum parsimony tree was estimated by using FastTree. Tree visualization was done using iTOL [29].

### Supplementary Information

**Additional  file 1:** Outbreak of CCPP in 12 goat and gazelle farms in Northern Oman 2019/2020.

**Additional file 2:** Clinical manifestations, pathological and histopathological findings in goats and gazelles from twelve farms in Northern Oman.

**Additional file 3:**  Sampling, isolation and PCR detection of *Mccp *in eleven goat and gazelle farms from Northern Oman.

**Additional file 4:** *Mycoplasma capricolum *subspecies* capripneumoniae* strains whose genomes were retrieved from Genbank (March 2022) and used in this study.

## Data Availability

The sequence data generated and analysed during the current study are available in the Genbank database (NCBI) in Bioproject No. PRJNA939501 (https://www.ncbi.nlm.nih.gov/sra/?term=PRJNA939501). Further datasets are included in this published article and its additional information files or available from the corresponding author on reasonable request.

## Abbreviations

CBPP: Contagious caprine pleuropneumonia
cgMLST: Core genome multi locus sequence typing
Cq: Quantification cycle
H&E: Hematoxylin and eosin stain
IHC: Immunohistochemistry
*Mccp*: *Mycoplasma capricolum* Subspecies *capripneumoniae*
MLST: Multi locus sequence typing
PCR: Polymerase chain reaction
SNP: Single nucleotide polymoprhism
UAE: United Arab Emirates
WOAH: World Organisation of Animal Health
