# Supplementary material for: A molecular epidemiological investigation of contagious caprine pleuropneumonia in goats and captive Arabian sand gazelle (Gazella marica) in Oman
Source: BMC Vet Res. 2024 Apr 25;20:155. doi: 10.1186/s12917-024-03969-1 (PMC11044429; doi:10.1186/s12917-024-03969-1)
Supplement: Supplementary file 4 — Additional file 4: Mycoplasma capricolum subspecies capripneumoniae strains whose genomes were retrieved from Genbank (March 2022) and used in this study. [file 12917_2024_3969_MOESM4_ESM.docx]

**Additional Table 4.** *Mycoplasma capricolum* subspecies *capripneumoniae* strains whose genomes were retrieved from Genbank (March 2022) and used in this study.

| **Strain** | **Country** | **Year** | **Host** | **Tissue** | **GenBank acc. No.** |
| --- | --- | --- | --- | --- | --- |
| ILRI181 | Kenya | 2012 | *Capra hircus* | pleural fluid | LN515399 |
| Bagamoyo | Tanzania | 2013 | *Capra hircus* | lung | CP041704 |
| 04012 | Qatar | 2004 | *Capra aegagrus* | pleural fluid | CP040917 |
| 438LP | Chad | 1994 | *Capra hircus* | lung | CP041708 |
| 05021 | Sudan | 2004 | *Capra hircus* | lung | CP041700 |
| 95043 | Niger | 1995 | *Capra hircus* | lung | CP041705 |
| C550/1 | UAE | 1991 | *Capra hircus* | lung | CP041703 |
| 12002 | Tajikistan | 2011 | *Capra hircus* | lung | CP041702 |
| M1601 | China | 2007 | *Capra hircus* | lung | CP017125 |
| 033C1 | Turkey | 2007 | *Capra hircus* | lung | CP041712 |
| 8991 | Oman | 1986 | *Capra hircus* | lung | CP041701 |
| 2/90 | Ethiopia | 1991 | *Capra hircus* | lung | CP041710 |
| Abomsa | Ethiopia | 1982 | *Capra hircus* | pleural fluid | LM995445 |
| C5 | Oman | 1994 | *Capra hircus* | lung | CP041709 |
| AMRC-C758 | Sudan | 1983 | *Capra hircus* | lung | CP041711 |
| F38 | Kenya | 1976 | *Capra hircus* | lung | LN515398 |
| Yatta/B | Kenya | 1997 | *Capra hircus* | lung | CP041707 |
| 97097-Erer | Ethiopia | 1997 | *Capra hircus* | lung | CP041706 |
| 87001 | China | 1958 | *Capra hircus* | lung | CP006959 |
| zly1309F | China | 2012 | *Pantholops hodgsonii* | lung | CP019061 |
